# Retroviral Restriction Factor APOBEC3G Delays the Initiation of DNA Synthesis by HIV-1 Reverse Transcriptase

**DOI:** 10.1371/journal.pone.0064196

**Published:** 2013-05-23

**Authors:** Madison B. Adolph, Jonathon Webb, Linda Chelico

**Affiliations:** Department of Microbiology and Immunology, University of Saskatchewan, Saskatoon, Saskatchewan, Canada; Centro de Biología Molecular Severo Ochoa (CSIC-UAM), Spain

## Abstract

It is well established that the cytosine deaminase APOBEC3G can restrict HIV-1 virions in the absence of the virion infectivity factor (Vif) by inducing genome mutagenesis through deamination of cytosine to uracil in single-stranded HIV-1 (−)DNA. However, whether APOBEC3G is able to restrict HIV-1 using a deamination-independent mode remains an open question. In this report we use *in vitro* primer extension assays on primer/templates that model (−)DNA synthesis by reverse transcriptase from the primer binding site (PBS) and within the protease gene of HIV-1. We find that APOBEC3G is able to decrease the initiation of DNA synthesis by reverse transcriptase approximately 2-fold under conditions where reverse transcriptase is in excess to APOBEC3G, as found in HIV-1 virions. However, the delay in the initiation of DNA synthesis on RNA templates up to 120 nt did not decrease the total amount of primer extended after extended incubation unless the concentration of reverse transcriptase was equal to or less than that of APOBEC3G. By determining apparent K_d_ values of reverse transcriptase and APOBEC3G for the primer/templates and of reverse transcriptase binding to APOBEC3G we conclude that APOBEC3G is able to decrease the efficiency of reverse transcriptase-mediated DNA synthesis by binding to the RNA template, rather than by physically interacting with reverse transcriptase. All together the data support a model in which this deamination-independent mode of APOBEC3G would play a minor role in restricting HIV-1. We propose that the deamination-independent inhibition of reverse transcriptase we observed can be a mechanism used by APOBEC3G to slow down proviral DNA formation and increase the time in which single-stranded (−)DNA is available for deamination by APOBEC3G, rather than a direct mechanism used by APOBEC3G for HIV-1 restriction.

## Introduction

Since APOBEC3G (A3G) was identified in 2002 [1], it has become known for its ability to restrict infectivity of HIV-1 virions in the absence of the virion infectivity factor (Vif) [2], [3], [4], [5], [6], [7]. A3G is a single-stranded (ss)DNA cytosine deaminase that induces cytosine (C) to uracil (U) deaminations of 5′CC or 5′CCC motifs (underlined C is deaminated) during synthesis of HIV-1 (−)DNA [8], [9]. This leads to inactivation of HIV-1 through guanine (G) to adenine (A) hypermutation of the virus genome strand from reverse transcriptase (RT) using uracil as a template during synthesis of (+)DNA [10], [11], [12].

A3G has two cytosine deaminase domains (CD), known as CD1 (N-terminal domain) and CD2 (C-terminal domain) [13]. Each domain co-ordinates Zn through His and Cys residues in the conserved consensus sequence His-X-Glu-X_23–28_-Pro-Cys-X_2–4_-Cys [14]. In A3G, only the CD2 is catalytically active [13]. The CD1 is able to bind nucleic acids and is necessary for incorporation of A3G into HIV-1 virions [13]. A3G can also form dimers, tetramers and higher order oligomers through the CD1 and CD2 and this is facilitated by binding to DNA or RNA [15], [16], [17], [18], [19], [20]. Deamination of cytosines on ssDNA by A3G occurs processively, meaning that multiple cytosine residues are deaminated in a single A3G-DNA encounter [21]. The processive motion of A3G is mediated by the CD1 and occurs by facilitated diffusion jumping and sliding events that appear to be required to effectively catalyze deamination on the HIV (−)DNA that is interspersed with RNA/DNA hybrid regions [16], [22].

The nucleocapsid protein (NC) and RT are the essential enzymes for synthesis of the HIV DNA provirus. The nucleic acid chaperone NC is required to unfold and anneal the host tRNA^Lys-3^ to the HIV Primer Binding Site (PBS) near the 5′-end of the genomic RNA (gRNA) [23]. This enables the RT to copy the gRNA to form the (−)DNA primer, termed the (−) strand strong stop DNA ((−)sssDNA) [24]. Then NC transfers the DNA/tRNA^Lys-3^ hybrid to the 3′-end of the gRNA for synthesis of the (−)DNA [24]. During (−)DNA synthesis, RT degrades the RNA using its RNaseH domain. These RNA fragments spontaneously dissociate or are displaced by RT [24]. This enables A3G to access ssDNA regions of the (−)DNA and deaminate C to U [9]. After completion of synthesis of the first DNA strand, the RT uses two RNaseH resistant polypurine tracts (PPT) in the gRNA to prime (+)DNA synthesis [25].

In addition to catalyzing deaminations, A3G may also physically inhibit RT-mediated DNA synthesis [26], [27], [28], [29] or other replicative functions such as NC-mediated strand annealing [30], [31] and RNaseH activity [32]. Termed the “deamination-independent” mode, studies have shown that this mode can decrease the accumulation of reverse transcripts up to ∼90% with late transcripts being reduced more than early transcripts [26], [28], [30], [32]. However, others have reported that A3G does not have a deamination-independent mode and that deamination is the only mechanism that A3G utilizes to restrict HIV-1 [33], [34], [35]. The doubts in the existence of a deamination-independent mode came from two lines of evidence. First, some research groups found that the decrease in the accumulation of HIV-1 reverse transcripts only occurred at a high transfection level of exogenous A3G into 293T cells [34], [35]. When transfection levels were decreased to mimic cellular levels of A3G the deamination-independent mode of inhibition was not observed [34], [35]. Second, catalytically inactive mutants of A3G such as E259Q or C291S have been used to test this mode and researchers have seen a 60–100% decline in the inhibition of reverse transcripts from that of native A3G, suggesting that deamination is necessary [28], [33], [34], [35]. However, there been little to no biochemical analyses of these mutants and they may not be true A3G proxies [28]. There are *in vitro*-based studies that have identified that A3G inhibits DNA synthesis by RT, but there is no consensus on a mechanism or methodologies [26], [28]. An earlier publication provided evidence that A3G bound the template to impede DNA elongation by reverse transcriptase [26]. However, a recent publication suggests that A3G can inhibit reverse transcription by physically binding to RT [29]. Further, it has been suggested that the biochemical basis of this inhibition is that A3G inhibits the processivity of RT, although the mechanism, i.e., whether A3G binds the template RNA or RT, was not proposed [27].

In this paper, we investigated the presence of a deamination-independent mode of inhibition of RT-mediated DNA synthesis by A3G. We show that A3G is able to inhibit initiation of RT polymerization at physiologically relevant ratios of reaction components where RT is in excess to the template and A3G. However, this inhibition which we observe under conditions where RT can interact with a primer/template (p/t) molecule at most once, is not sufficient to inhibit the overall amount of polymerization in an extended time course where RT can interact with the same p/t multiple times. As a result, although A3G can decrease the polymerization rate of RT by delaying primer initiation, we conclude that the deamination-independent effects of A3G are not likely to be dominant over a deamination-dependent mode of HIV-1 restriction.

## Materials and Methods

### Molecular cloning and synthesis of RNA templates

Molecular cloning primers were obtained from Integrated DNA Technologies and are listed in Table S1.

For the PBS RNA template, a 106 nucleotide (nt) segment near the 5′-end of the HIV-1 genome (nt 571–674) encompassing the PBS and upstream region was PCR amplified. For the protease RNA template, a 120 nt segment of the HIV-1 genome (2282–2401 nt) was PCR amplified. The PCR amplicons were cloned into the BamHI and EcoRI sites of the pSP72 vector (Promega). Sequences were amplified from the HIV-1 clone 93th253.3 (GenBank accession number U51189) obtained through the AIDS Research and Reference Reagent Program, Division of AIDS, NIAID, National Institutes of Health; p93th253.3 was from Dr. Feng Gao and Dr. Beatrice Hahn [36]. The sequences were verified and then used for synthesis of RNA template by linearizing the vector at the BamHI site and using it as a T7 RNA polymerase substrate according to manufacturer's instructions for the Ambion Megascript kit.

Site-directed mutagenesis to construct the A3G E259Q clone was conducted using the QuikChange site-directed mutagenesis protocol (Agilent Technologies) with the pAcG2T-A3G vector as the template [21].

### Protein Expression and Purification

Recombinant baculovirus for expression of GST-NC, GST-A3G, and GST-A3G E259Q was constructed as described previously [21], [22]. *Sf*9 cells were infected with recombinant NC or A3G virus at a multiplicity of infection of 1 and harvested after 72 h. Cells were lysed and purified as described previously to obtain NC or A3G protein cleaved from the GST tag [16], [22]. Cleaved protein fractions were stored at −80°C. The NC and A3G forms are ∼95% pure. HIV-1 RT p66/p51 [37] was generously provided by Dr. Stuart F. J. Le Grice (NCI, National Institutes of Health).

### Primer extension assays

The 106 nt template RNA containing the PBS (nt 571–674) was heat annealed to an 18 nt ^32^P-labeled RNA primer to mimic tRNA^Lys,3^ primer binding or a 24 nt ^32^P-labeled RNA/DNA primer to mimic a tRNA^Lys,3^ primer that had been extended by 6 nt. In addition, an RNA template comprising part of the HIV-1 protease (nt 2282–2401) was used with a heat annealed 20 nt ^32^P-labeled DNA primer. Primers were obtained from Integrated DNA Technologies and are listed in Table S1. The p/t (10 nM) was then used in reactions containing RT buffer (50 M Tris, pH 7.5, 40 mM KCl, 10 mM MgCl_2_, 1 mM DTT), 200 µM dNTPs, 175 nM NC, and 40, 120, or 480 nM RT in the absence or presence of A3G (40, 80, 160, 320 or 480 nM) or A3G E259Q (40 or 480 nM). The 40 nM concentration of A3G was used to obtain a 4∶1 ratio of enzyme to the p/t to mimic the predicted molar ratio of A3G to the RNA genomes in a *ΔVif* HIV virion [24], [38], [39], [40]. Reactions were preincubated at 37°C for 1 min before the addition of dNTPs which were used to start the reaction. A negative control was used which contained all reaction components except reverse transcriptase to ensure there was no contaminating polymerase activity. In experiments where a heparin sodium trap was used (1 mg/mL) to ensure single-turnover conditions [41], reactions were preincubated at 37°C for 1 min before the addition of a dNTP and heparin sodium mixture equal in volume to the preheated reaction components. Reactions were stopped by adding a 5-fold excess of 20 mM EDTA and 95% formamide. Primer extension was visualized by resolving samples on a 16% denaturing 8 M urea polyacrylamide gel. Gel band intensities were measured by phosphorimaging with a Bio-Rad FX scanner. The integrated gel band intensities of all bands in a lane were calculated with ImageQuant software (GE Healthcare) and used to determine the relative amounts of extended and unextended primer. The background signal intensity for each gel was calculated using the negative control lane. Statistical significance of primer extension assay results was determined using an unpaired t-test.

### Steady state rotational anisotropy assays

Protein-p/t and protein-protein binding measurements were made by monitoring changes in steady-state fluorescence depolarization (rotational anisotropy) [42]. For measuring binding to the p/t, three 5′-end fluorescein (F)-labeled primers corresponding to those used in the primer extension assays were heat annealed to their corresponding RNA template (PBS RNA or protease RNA, Table S1) to form the binding substrate. For measuring binding of RT to A3G, a fluorescein labeled A3G (F-A3G) was obtained by using a Fluorescein-EX labeling kit (Invitrogen), according to manufacturer's instructions.

The rotational anisotropy experiments (60 µL) were incubated at 21°C and contained RT buffer, p/t or F-A3G (50 nM) and increasing concentrations of RT or A3G. For competition experiments, 200 nM of A3G was prebound to 50 nM of p/t. Protein concentrations used ranged from 0–5 µM for measuring p/t binding affinities and 0–11 µM for measuring F-A3G binding affinity. Rotational anisotropy was measured with a QuantaMaster QM-4 fluorometer (Photon Technology International). Samples were excited with vertically polarized light at 495 nm (6 nm band pass), and both vertical and horizontal emissions were monitored at 520 nm (6 nm band pass). Apparent dissociation constants (K_d_) were determined through regression analysis using Sigma Plot 11.2 software.

## Results

### Inhibition of RT-mediated primer extension by A3G

**Figure 1:**
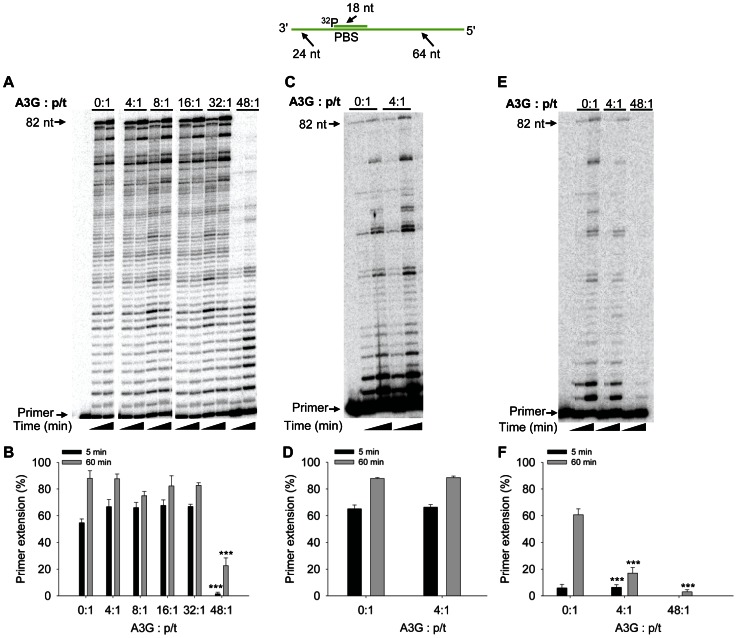
RT-mediated primer extension from the HIV-1 PBS in the presence of A3G. An 18 nt ^32^P-labeled RNA primer containing a sequence complementary to the HIV-1 PBS was annealed to a 106 nt RNA containing the PBS (sketch). Complete extension of the primer results in a product of 82 nt (sketch). The p/t was used at a concentration of 10 nM. (A) Primer extension by RT (480 nM) in the absence (0∶1) or presence (4∶1, 8∶1, 16∶1, 32∶1, 48∶1) of increasing amounts of A3G relative to the p/t concentration. Reactions were sampled at 5 and 60 min. (B) Quantification of primer extension (%) from gels shown in panel A for 5 min (black bars) and 60 min (grey bars). (C) Primer extension reactions carried out as described for (A), but in the absence of the NC protein. (D) Quantification of primer extension (%) from gel shown in panel C for 5 min (black bars) and 60 min (grey bars). (E) Primer extension by RT (120 nM) in the absence (0∶1) or presence (4∶1, 48∶1) of increasing amounts of A3G relative to the p/t concentration. Reactions were sampled at 5 and 60 min. (F) Quantification of primer extension (%) from gel shown in panel E for 5 min (black bars) and 60 min (grey bars). Primer extension (%) values are averaged from three independent trials. Designations for significant difference from 0∶1 values were *p*≤0.001 (***), *p*≤0.01(**), or *p*≤0.05 (*).

Primer extension by RT from the PBS is the initial step in HIV-1 proviral formation. The initiation of synthesis from this site is inefficient due to the orientation with which RT binds the RNA primer [43] and could be the most vulnerable target. This suggests that A3G inhibition of RT polymerization, if occurring, could be best observed using the PBS region. Based on previous reports on the number of molecules of A3G and RT encapsidated in HIV-1 particles we calculated the expected molar ratio of each of these components to the RNA genomes [24], [39]. Using these ratios in which RT is in excess to A3G (12∶1) and the p/t (48∶1), we carried out primer extension assays (Figure 1A) in the presence of saturating amounts of NC protein (1 NC/7 nt). We found that RT could extend almost all of the primer within 60 min and that addition of A3G at 4∶1 to the p/t caused no decrease in overall primer extension by RT (Figure 1A–B, compare 0∶1 and 4∶1). Increasing the A3G concentration did not increase its inhibitory activity until the A3G concentration reached a 48-fold excess to the p/t (Figure 1A–B, 48∶1). At a 48-fold excess of A3G to the RNA template, and at an equal amount to RT, there was a decrease in overall primer extension (Figure 1A–B). The decrease in extension products was highest at the earlier time point (Figure 1A–B, 5 min, 37-fold) than the later time point (Figure 1A–B, 60 min, 4-fold). These results suggest that the ratios of reaction components are critical in determining whether A3G can inhibit RT-mediated primer extension. We also tested the effect of NC protein since our reactions contain saturating amounts. The NC protein may be able to decrease A3G-mediated inhibition of RT polymerization since A3G and NC protein can bind the same RNA substrate [44]. At the low concentration of A3G (4∶1 to the p/t), we found that the absence of NC protein did not alter RT polymerization or the effect of A3G on RT (compare Figure 1A–B and Figure 1C–D, 0∶1 and 4∶1). Therefore, in all other reactions we maintained the use of saturating amounts of NC protein (1 NC/7 nt).

To further investigate the extent to which A3G can inhibit RT polymerization we used the same p/t as in Figure 1A, but conducted the experiment with only a 12-fold excess of RT to the p/t and a 4-fold excess of A3G to the p/t (Figure 1E). Here we find that A3G at a ratio of 4∶1 to the p/t is able to inhibit primer extension by RT (Figure 1E–F, 3.5-fold, 60 min). At a 48-fold excess of A3G to the p/t, A3G can virtually block any initiation of RT-mediated primer extension (Figure 1E–F, 48∶1). These results further demonstrate that the ratio of reaction components is important in determining the extent to which A3G can inhibit RT polymerization.

**Figure 2 pone-0064196-g002:**
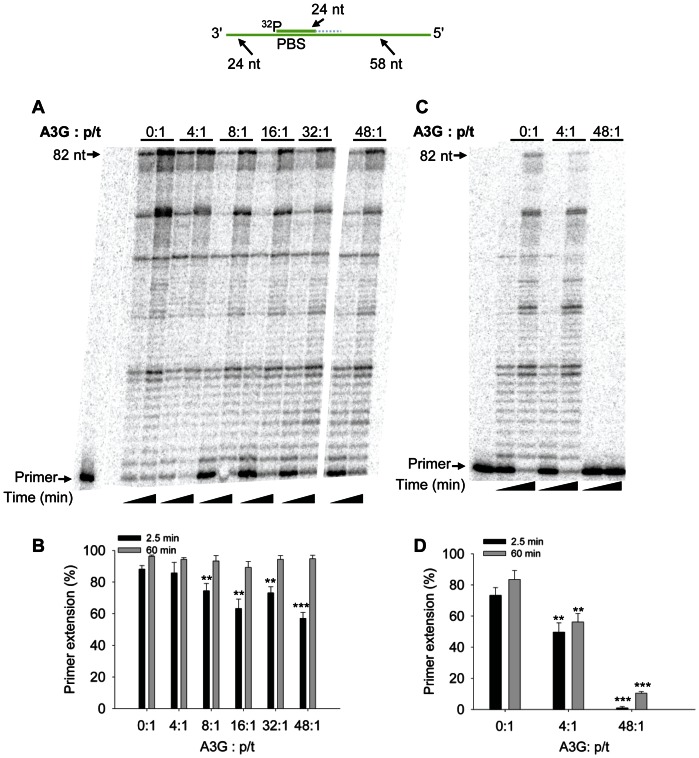
Decreased A3G-mediated inhibition of RT polymerization from a partially extended RNA primer. A 24 nt ^32^P-labeled RNA/DNA primer containing the RNA sequence complementary to the HIV-1 PBS and 6 DNA bases to mimic partial extension by RT was annealed to a 106 nt RNA containing the PBS (sketch). Complete extension of the primer results in a product of 82 nt (sketch). The p/t was used at a concentration of 10 nM. (A) Primer extension by RT (480 nM) in the absence (0∶1) or presence (4∶1, 8∶1, 16∶1, 32∶1, 48∶1) of increasing amounts of A3G relative to the p/t concentration. Reactions were sampled at 2.5 and 60 min. (B) Quantification of primer extension (%) from gels shown in panel A for 2.5 min (black bars) and 60 min (grey bars). (C) Primer extension by RT (120 nM) in the absence (0∶1) or presence (4∶1, 48∶1) of increasing amounts of A3G relative to the p/t concentration. Reactions were sampled at 2.5 and 60 min. (D) Quantification of primer extension (%) from gel shown in panel C for 2.5 min (black bars) and 60 min (grey bars). Primer extension (%) values are averaged from three independent trials. Designations for significant difference from 0∶1 values were *p*≤0.001 (***), *p*≤0.01(**), or *p*≤0.05 (*).

**Figure 3:**
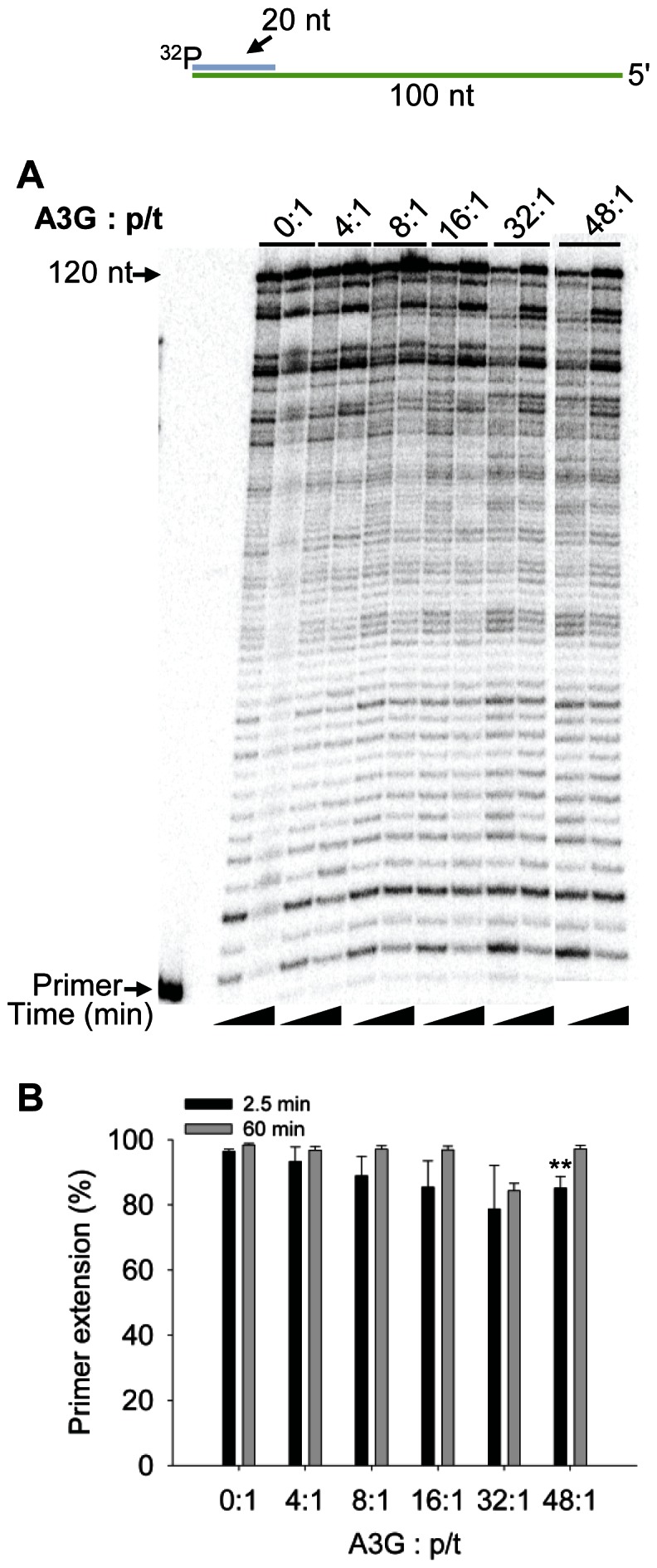
RT polymerization from a DNA primer is less susceptible to A3G-mediated inhibition. A 20 nt ^32^P-labeled DNA primer was annealed to a 120 nt RNA containing part of the HIV-1 protease gene (sketch). Complete extension of the primer results in a DNA of 120 nt (sketch). The p/t was used at a concentration of 10 nM. (A) Primer extension by RT (480 nM) in the absence (0∶1) or presence (4∶1, 8∶1, 16∶1, 32∶1, 48∶1) of increasing amounts of A3G relative to the p/t concentration. Reactions were sampled at 2.5 and 60 min. (B) Quantification of primer extension (%) from gels shown in panel A for 2.5 min (black bars) and 60 min (grey bars). Primer extension (%) values are averaged from three independent trials. Designations for significant difference from 0∶1 values were *p*≤0.001 (***), *p*≤0.01(**), or *p*≤0.05 (*).

To determine whether A3G could inhibit RT polymerization on a p/t which is more efficiently extended than the PBS p/t, we annealed a partially extended PBS primer to the PBS RNA template. The 24 nt RNA/DNA primer was synthesized with 18 nt of RNA to bind the PBS and 6 nt of DNA on the 3′-end to mimic a partially extended tRNA^Lys,3^ (Figure 2, sketch). The “PBS+6” p/t facilitates polymerization by RT, since RT interacts with the PBS+6 primer similarly to a DNA primer [43]. As expected, we observed more efficient primer extension from the PBS+6 p/t compared to the PBS p/t (compare Figure 1A–B and Figure 2A–B, 0∶1). On the PBS+6 p/t, we also found that A3G could not inhibit RT-mediated primer extension at a concentration that was 4-fold in excess to the primer (Figure 2A–B). Only when A3G was added 8-, 16-, 32- or 48- fold in excess to the p/t did we observe some inhibition of primer extension at the early time point (Figure 2A–B, 2.5 min). The primer extension was inhibited at most 1.2- to 1.5- fold at 2.5 min, but by 60 min the inhibition of primer extension was not observable (Figure 2A–B). Comparing 48-fold excess A3G conditions, we observe less A3G-mediated inhibition of RT polymerization on the PBS RNA template with the PBS+6 primer than the PBS primer (Figure 1A–B, 5 min, compare 37-fold inhibition to Figure 2A–B, 2.5 min, 1.5-fold inhibition; p-value 0.0001). When the concentration of RT was lowered 4-fold, a low concentration of A3G (4∶1 ratio to p/t) was able to decrease RT-mediated primer extension 1.5-fold and a high concentration of A3G (48∶1 ratio to p/t) could in effect block all RT-mediated primer extension (Figure 2C–D). These data are consistent with previous RT literature that primer extension is more efficient from a DNA primer [45], [46], but if the RT concentration is lowered it appears that A3G can cause inhibition of RT polymerization. To confirm these results we examined a second primer that was completely composed of DNA nucleotides. This primer was annealed to 120 nt of the protease (+)RNA. On this p/t we found that A3G was unable to cause any substantial inhibition of RT from concentrations that ranged from 4- to 48-fold in excess of the p/t (Figure 3A–B). These data confirm that extension from a DNA primer is less likely to be inhibited by A3G (compare Figure 2A–B and Figure 3A–B) than extension from an RNA primer (Figure 1A–B). All together, the results demonstrate that the nature of the p/t and therefore the ability of RT to effectively interact with the p/t [43] is a strong determining factor in the ability of A3G to inhibit RT polymerization.

### RNA template binding by A3G inhibits RT-mediated primer extension

We next investigated how A3G was inhibiting initiation of primer extension by RT. It has been suggested that A3G can inhibit RT by binding to the template [26] or RT itself [29]. Our results indicate that A3G binding to the RNA template is of importance since alterations of the p/t type to one that is more efficiently extended can alter the effect of A3G on RT (compare Figure 1A–B, Figure 2A–B, and Figure 3A–B). To confirm this we used rotational anisotropy to determine apparent dissociation constants (K_d_) of A3G and RT binding to the different p/t types used in our experiments. Consistent with previous reports for RT [47], [48] and our primer extension data (Figures 1–3), we found that RT bound an RNA template with an RNA primer (Table 1, PBS p/t, K_d_ of 2523 nM) with less affinity than a partially extended primer (Table 1, PBS+6 p/t, K_d_ of 581 nM) or DNA primer (Table 1, protease p/t, K_d_ of 442 nM). A3G bound 3-fold tighter to the PBS p/t than RT (Table 1, K_d_ of 778 nM) and bound the partially extended PBS+6 p/t with 1.6-fold greater affinity than RT (Table 1, K_d_ of 354 nM). A3G bound less well (∼1.6-fold) to the protease p/t than RT (Table 1, compare K_d_ values of 1014 nM and 442 nM, respectively).

**Table 1:** Apparent dissociation constants (K_d_) of RT, A3G, and A3G E259Q from primer/templates.

	K_d_ (nM)		
Enzyme	PBS p/t	PBS+6 p/t	protease p/t
RT	2523±231	581±31	442±20
A3G	778±17	354±21	1014±99
E259Q	1097±109	685±89	1799±214
RT+A3G		981±55	479±20

**Figure 4:**
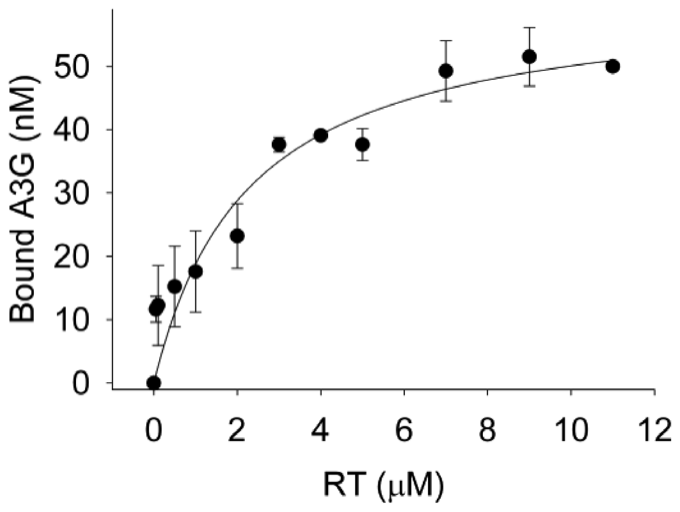
Analysis of the interaction between RT and fluorescently labeled A3G using steady-state fluorescence depolarization. The apparent dissociation constant (K_d_) was determined by titrating increasing concentrations (*x-axis*) of RT to 50 nM of fluorescein (F)-labeled A3G. RT binds F-A3G with an apparent K_d_ of 2.3±0.8 µM. Fraction of F-A3G bound is calculated from anisotropy values averaged from three independent trials.

Additionally, we used fluorescently labeled A3G (F-A3G) as a binding substrate for RT during rotational anisotropy experiments to test if RT and A3G could interact. We found that the two enzymes can interact with an apparent K_d_ of 2300 nM (Figure 4). This interaction is at least 4-fold lower than the affinity of RT for the p/t types with DNA primers (Table 1, 4-fold (PBS+6 p/t) to 5-fold (protease p/t)), suggesting that RT is more likely to bind these p/t molecules than A3G. A3G has at least a 2-fold lower affinity for RT than all three p/t types (Table 1, 2-fold (protease p/t), 3-fold (PBS p/t), 6-fold (PBS+6 p/t)), suggesting that A3G is also more likely to bind the RNA template than RT.

**Figure 5:**
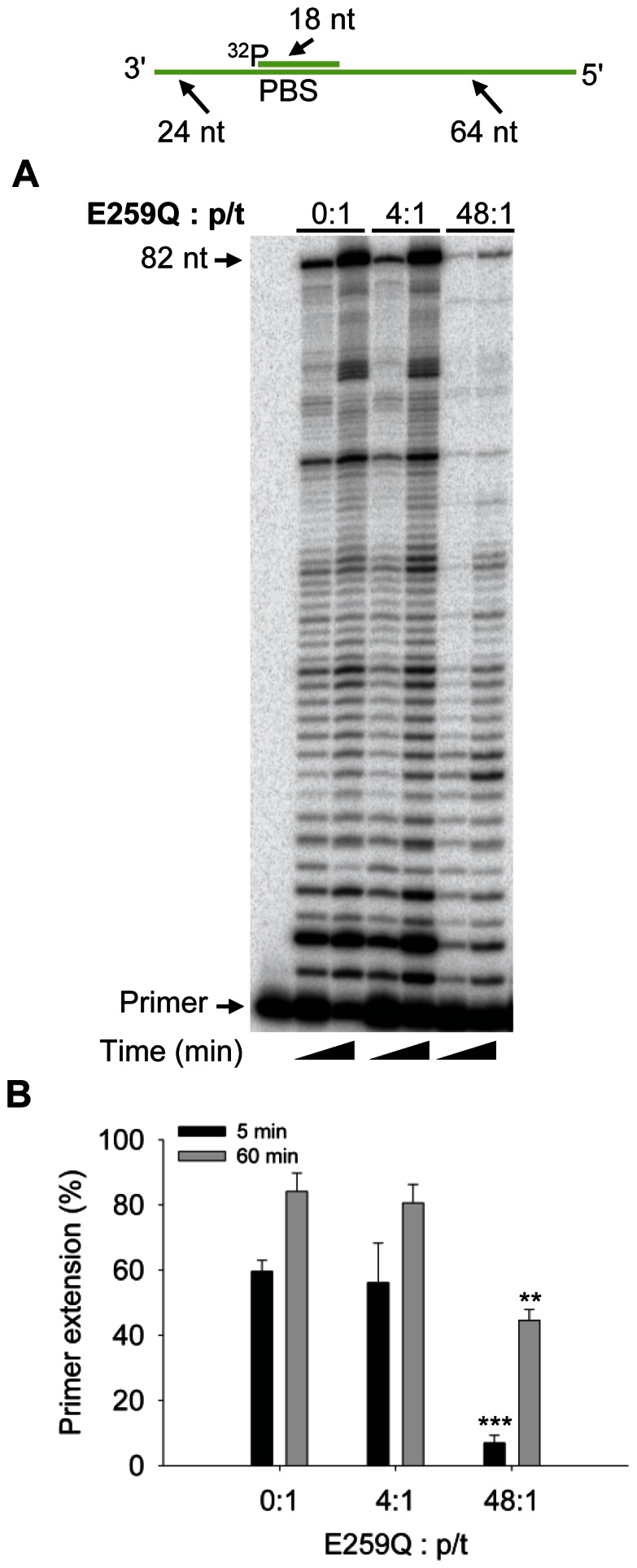
A3G E259Q inhibition of RT-mediated primer extension from the HIV-1 PBS. An 18 nt ^32^P-labeled RNA primer containing a sequence complementary to the HIV-1 PBS was annealed to a 106 nt RNA containing the PBS (sketch). Complete extension of the primer results in a product of 82 nt (sketch). The p/t was used at a concentration of 10 nM. (A) Primer extension by RT (480 nM) in the absence (0∶1) or presence (4∶1, 48∶1) of A3G E259Q relative to the p/t concentration. Reactions were sampled at 5 and 60 min. (B) Quantification of primer extension (%) from gel shown in panel A for 5 min (black bars) and 60 min (grey bars). Primer extension (%) values are averaged from three independent trials. Designations for significant difference from 0∶1 values were *p*≤0.001 (***), *p*≤0.01(**), or *p*≤0.05 (*).

However, for the PBS p/t, RT had a similar apparent K_d_ from the p/t and A3G (Table 1 and Figure 4, K_d_ values of 2523 nM and 2300 nM, respectively). Despite binding data suggesting that A3G is more likely to bind to the PBS p/t than RT (compare Table 1 and Figure 4), to exclude that there may be a combination of both protein-protein and protein-nucleic acid interactions occurring we used a mutant A3G that bound the p/t molecules with less affinity than wild-type A3G. If protein-protein interactions between RT and A3G are necessary then we would expect to see no difference between wild-type A3G and an A3G mutant that bound RNA with less affinity. We have found that the E259Q mutant of A3G (referred to as E259Q), although often used as a proxy for wild-type A3G, bound all p/t molecules used in our study 1.4- to 2-fold less than A3G (Table 1). This also corresponded to a 2- to 5-fold decrease in the ability of E259Q to inhibit RT polymerization on the PBS p/t compared to A3G (compare Figure 5A–B and Figure 1A–B, 48∶1, 5 min, 5-fold difference (p-value 0.0233) and 48∶1, 60 min, 2-fold difference (p-value 0.0048)). Overall the data support a model in which the affinity of A3G and RT for the p/t molecules influences the ability of A3G to inhibit RT-mediated polymerization with the physical interaction of A3G and RT (Figure 4) having no discernible effect.

### Mechanism of A3G-mediated inhibition of RT

**Figure 6:**
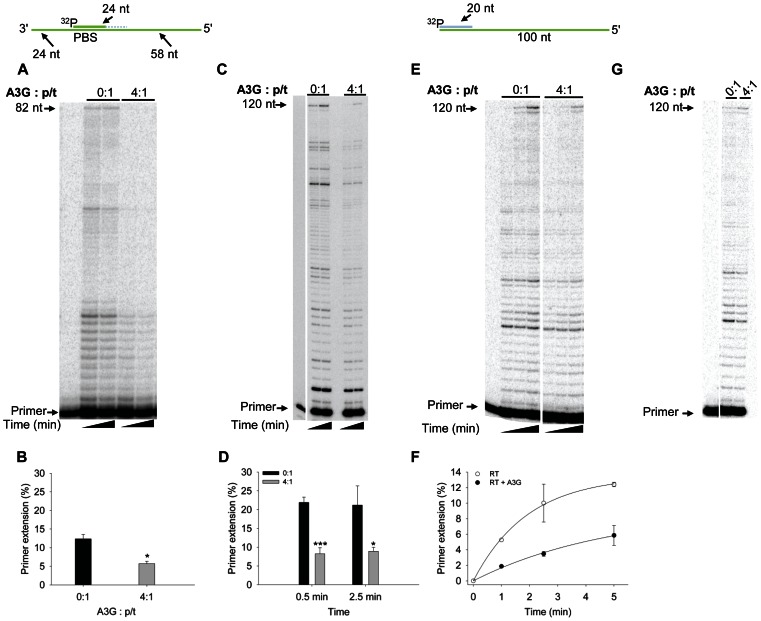
A3G inhibits the rate of primer extension but not the processivity of RT. The p/t was used at a concentration of 10 nM. (A) A 24 nt ^32^P-labeled RNA/DNA primer containing the RNA sequence complementary to the HIV-1 PBS and 6 DNA bases to mimic partial extension by RT was annealed to a 106 nt RNA containing the PBS (sketch on left). Complete extension of the primer results in a product of 82 nt (sketch on left). Primer extension by RT (480 nM) in the presence of a heparin trap and the absence (0∶1) or presence (4∶1) of A3G, indicated relative to p/t concentration. Reactions were sampled at 1 min and 5 min. (B) Quantification of primer extension (%) after 1 min from gel shown in panel A for A3G∶p/t ratios of 0∶1 (black bar) and 4∶1 (grey bar). (C) A 20 nt ^32^P-labeled DNA primer was annealed to a 120 nt RNA containing part of the HIV-1 protease gene (sketch on right). Complete extension of the primer results in a DNA of 120 nt (sketch on right). Primer extension by RT (480 nM) in the presence of a heparin trap and the absence (0∶1) or presence (4∶1) of A3G, indicated relative to p/t concentration. Reactions were sampled at 0.5 min and 2.5 min. (D) Quantification of primer extension (%) from gels shown in panel C for 0.5 min (black bars) and 2.5 min (grey bars). (E) Primer extension by RT (40 nM) on the protease p/t (sketch on right) in the absence (0∶1) or presence (4∶1) of A3G, indicated as relative to the p/t concentration. Reactions were sampled at 1, 2.5 and 5 min. (F) Quantification of primer extension (%) from gels shown in panel E for RT in the absence (open circles) or presence of A3G (black circles). The primer extension rate of RT (4.6%/min) is decreased 3-fold in the presence of A3G (1.5%/min). (G) Comparison of RT-mediated primer extension reactions in the absence (0∶1) or presence (4∶1) of A3G where only 5% of the p/t was extended to ensure single-hit conditions. Under these conditions the size of the extension products can be used to determine the processivity of RT. Primer extension (%) values are averaged from three (A–D) or two (E–F) independent trials. Designations for significant difference from 0∶1 values were *p*≤0.001 (***), *p*≤0.01(**), or *p*≤0.05 (*).

In order to understand the underlying biochemical mechanism in which A3G can inhibit RT polymerization, we investigated how A3G affects RT polymerization per single interaction of RT with the p/t. To assess what is occurring between A3G and RT molecules in a single interaction with a p/t, we conducted a primer extension assay in the presence of a heparin trap and used conditions where RT is in excess (48∶1) to the p/t. In these reactions, RT and A3G are prebound to the p/t before the addition of dNTPs and the heparin trap. Since RT and A3G both bind the heparin trap, as soon as the enzymes dissociate from the p/t they will be unable to rebind any additional p/t molecules (data not shown). As a result, this assay can measure the processive ability of RT, i.e., the number of nucleotides that RT can incorporate in a single encounter with a p/t molecule, and the efficiency of initiating DNA synthesis from a primer. To see RT-mediated primer extension in this type of reaction we used the two p/t types on which RT is processive, the PBS+6 p/t and the protease p/t. On the PBS+6 p/t we found that A3G could inhibit initiation of DNA synthesis 2-fold (Figure 6A–B). This demonstrates that more RT molecules dissociate from the RNA template before extending the primer in the presence of A3G than in its absence (Figure 6A–B). With increased time, both the RT alone and RT+A3G reactions did not increase in the amount of fully extended primer, demonstrating the low processivity on this substrate (Figure 6A, compare 82 nt band).

We next focused on the protease p/t for which RT is the most proficient at extending (Figure 3A–B). On the protease p/t, we found that A3G decreased initiation of DNA synthesis by 2.5-fold (Figure 6C–D), similar to the PBS+6 p/t (Figure 6A–B). However, although the amount of protease primer extended remains the same for 0.5 min and 2.5 min of the reaction (Figure 6D, 0∶1 and 4∶1), the amount of final product increases (Figure 6C, compare 120 nt band) in the absence and presence of A3G indicating that RT molecules that remain bound to the p/t are able to completely copy the RNA template in a single p/t encounter. These results suggest that A3G inhibits or delays initiation of DNA synthesis by RT as opposed to inhibiting RT processivity.

To confirm these results, we used another experimental system that enables the observation of single interactions of p/t and RT. In the absence of a trap we can achieve single-hit kinetics by keeping RT-mediated primer extension below 15% [49], [50]. Although enzymes could interact with many substrates since the experiment is conducted under steady-state, by keeping the p/t usage low (<15% p/t extended) we can decrease the chance of a p/t molecule being encountered twice in the reaction by an RT molecule to negligible based on Poisson statistics [49], [50]. In addition, these single-hit conditions would enable the observation of the polymerization rate and processivity of RT. To achieve these conditions, we slowed down the reaction by using a 4∶1 ratio of RT to the protease p/t. The A3G molar concentration was in 4-fold excess to the RNA template (Figure 6E). Even under these conditions of lowered RT, we still observe primer extension in the presence of A3G (Figure 6E). However, the rate of primer extension is decreased by 3-fold, providing further evidence that A3G is delaying initiation of primer extension (Figure 6F). To determine whether A3G can also inhibit the processivity of RT, we compared reactions done under single-hit conditions with similar amounts of primer extension and determined whether the presence of A3G can decrease the size of extension products formed (Figure 6G). Comparisons of processivity must be done using reactions that have similar amounts of primer extended in order to directly compare whether a difference in the size of the extension products formed is due to *bona fide* changes in processivity or simply by a difference in the overall rate of primer extension. We observed no decrease in the size of the extension products when comparing reactions with similar amounts of primer extended demonstrating that A3G does not decrease the number of nucleotides that RT can incorporate per enzyme-substrate encounter (Figure 6G), but only slows down initiation of synthesis (Figure 6E–F).

To determine whether A3G inhibits RT from binding the RNA template or prevents RT from accessing the p/t junction as a mechanism for blocking initiation of primer extension we used rotational anisotropy. Specifically, we prebound a 4∶1 ratio of A3G to the p/t (PBS+6 or protease) and titrated in RT to determine its steady-state binding affinity for p/t types under these competitive conditions (Table 1, RT+A3G, PBS+6 p/t and protease p/t). We observed a 2-fold change in the apparent K_d_ of RT for the PBS+6 p/t (Table 1, compare K_d_ values of 581 nM (RT) and 981 nM (RT+A3G)) demonstrating that A3G can effectively compete for and partially block RT from binding the p/t. However, for the protease p/t, we observed no change in the RT binding affinity (Table 1, compare K_d_ values of 442 nM (RT) and 479 nM (RT+A3G)), despite observing a 2.5-fold decrease in the ability of RT to initiate DNA synthesis in the presence of a 4∶1 ratio of A3G to the p/t (Figure 6C–D). Since RT molecules bind the template strand and must undergo 1-dimensional sliding to locate the 3′OH at the p/t junction [51], the data suggest that on the protease p/t it is this search process that A3G disrupts, rather than inhibiting RNA template binding by RT. These two different mechanisms employed by A3G can be explained by the relative binding affinities of A3G and RT for the p/t types (Table 1). On the PBS+6 p/t, A3G has a 1.6-fold stronger binding affinity than RT. On the protease p/t, RT binds 2-fold tighter than A3G (Table 1). All together the data support a model in which the mechanism exerted by A3G is determined by its ability to effectively compete with RT for p/t binding. In the case where A3G binds with more affinity, the binding of RT to the p/t can be partially prevented by A3G (Table 1, RT+A3G, PBS+6). If RT binds with more affinity, A3G can bind concurrently with RT, as evidenced by a biochemical effect of A3G on RT polymerization (Figure 6C–D), and inhibits the ability of RT to locate the 3′OH at the p/t junction.

## Discussion

We conducted this research to gauge the contribution of the deamination-independent mode of A3G inhibition of RT and identify a mechanism. Since relative levels of RT and A3G in HIV-1 particles would predict that RT is always in excess to A3G [24], [38], [39], [40] we conducted our reactions with these relative ratios. Under these conditions and where RT and A3G can interact with a given p/t molecule multiple times, A3G has no significant effect on total primer extension (Figure 1A–B, Figure 2A–B and Figure 3A–B, compare 0∶1 and 4∶1) supporting the conclusion that the deamination-independent inhibition of RT is not a dominant mechanism used by A3G to restrict HIV-1. Nonetheless, it appears that per single interaction of RT with DNA, A3G can decrease the rate of initiation of primer extension (Figure 6). Thus, there may be a cumulative effect of delaying DNA synthesis at various primer termini on longer RNA templates that we could not observe on our *in vitro* substrates. This would explain how late transcripts are inhibited more than early transcripts in HIV-1 infected cells [26], [28], [30], [32].

The mechanism of inhibition of RT polymerization by A3G supported by our data is that A3G binds to the RNA template and either decreases the affinity of RT for the template by competitive binding (Table 1, RT+A3G, PBS+6 p/t) or decreases the efficiency with which RT locates primer 3′OH termini (Table 1, RT+A3G, protease p/t). Since A3G prefers to bind RNA or ssDNA compared with RNA/DNA hybrids or dsDNA [15], [21], [44], it is not likely that A3G directly blocks RT from binding a p/t junction in which RNA/DNA hybrids or dsDNA would be encountered, but that it can block initiation of synthesis from the primer by binding downstream on the RNA template. Specifically, since RT binds the template randomly and then uses 1-dimensional scanning to locate a 3′OH at the primer terminus [51], the RNA template-bound A3G could act as a scanning block so that RT is more likely to dissociate before it finds a p/t junction (Figure 6A–D). The likelihood of RT inhibition may increase with A3G concentration since this also would increase the degree of A3G oligomerization on the RNA template [15], [19]. This reasoning provides an explanation for why we only observed complete A3G-mediated inhibition of primer initiation at high concentrations of A3G relative to RT and the p/t (Figure 1E–F and Figure 2C–D). A cumulative effect could result on longer templates since at each dissociation and reassociation of RT *in vivo*, it is conceivable that A3G could potentially inhibit RT “re”-initiation.

Of particular note is that we did not find E259Q to be a suitable proxy for wild-type A3G. Although the E259Q catalytic mutant has been used when a deamination null mutant of A3G was required [33], [35], we found that E259Q did not bind RNA with as high affinity as wild-type A3G (Table 1) and this had a functional consequence (Figure 5A–B). This is in agreement with Bishop *et al.* [28] that found although the A3G E259Q mutant could inhibit HIV-1 reverse transcripts, it was to a lesser degree than wild-type A3G. Since our *in vitro* assay systems used RNA templates, which are not an A3G catalytic substrate [9], the DNA deamination activity of A3G appears to be inconsequential to the mechanism of RT inhibition characterized here. Rather the difference in the inhibitory potential of E259Q and wild-type A3G appears to be due to a biochemical difference in their RNA binding abilities (Table 1). We were able to use this difference in RNA binding abilities as a tool to extend our binding data and confirm that A3G inhibits RT polymerization by binding to the RNA template (Table 1 and Figure 5A–B).

This conclusion is further confirmed when we examine the protease p/t for which A3G had the lowest binding affinity (Table 1). When the RT concentration was lowered (Figure 6E–F) the reaction rate in the absence of A3G was also decreased (compare with Figure 3A–B) which demonstrates that the RT is no longer saturating all the p/t molecules. That an equal concentration of A3G to RT at the high RT concentration (Figure 3A–B, 48∶1, 2.5 min, <1.2-fold decrease in primer extension) did not produce the same results as an equal concentration of RT to A3G at the low RT concentration (Figure 6E–F, 4∶1, 2.5 min, 2.9-fold decrease in primer extension) supports the hypothesis that it is not the protein-protein interaction that is primarily resulting in inhibition of RT on the protease p/t, but A3G binding to the RNA template. Otherwise, we would expect that when A3G and RT are at equal concentrations the protein-protein interaction would result in the same degree of inhibition of RT-mediated polymerization since most RT molecules would have a bound A3G. Instead, it appears that the saturation of RT for the p/t is the most important factor which supports the hypothesis that A3G binding the RNA template results in inhibition of RT-mediated polymerization. Similar results were also obtained on the PBS p/t where a 1∶1 RT∶A3G concentration of 480 nM produced a 4-fold inhibition of primer extension (Figure 1A, 48∶1, 60 min), but a 1∶1 RT∶A3G concentration of 120 nM produced a 20-fold inhibition of primer extension (data not shown).

Our data are in agreement with reports that suggest A3G exerts it inhibitory activity on RT by binding to the template [26], [28]. Although there are points presented in this work which are not in agreement with other reports, this appears to be due to different experimental systems or different interpretation of results. Our data contrast similar *in vitro* experiments from Iwatani *et al.* [26] that tested A3G-mediated inhibition of polymerization by RT on synthetic substrates and found that A3G could cause significant inhibition of RT. However, in the study by Iwatani *et al.*, RT was used at a ratio of 1∶1 to the p/t and the molar concentration was 4- to 16-fold less than the A3G concentration [26]. By altering the ratio of A3G and RT, we could arrive at results that are consistent with what Iwatani *et al.* [26] found for A3G mediated inhibition (Figure 1E–F), which indicates that the inhibitory mechanism imparted by A3G is highly dependent on reaction components. However, due to our experimental conditions that attempt to model conditions in the HIV-1 virion in which RT is in excess to A3G (Figure 1A–B), our results indicate that the inhibition of RT by A3G is not as pronounced as Iwatani *et al.* concluded [26]. A recent study by Gillick *et al.* in primary cells and with physiological levels of exogenously produced A3G in 293T cells, was able to show that the length of individual HIV-1 reverse transcripts were decreased in the presence of A3G [27]. Gillick *et al.* [27] that suggested A3G affects RT processivity since the lengths of the (−)DNA measured at different times post-HIV-1 infection were shorter in the presence of A3G. In our system, we find no *bona fide* decrease in processivity when comparing reactions without or with A3G that have the same amount of primer extended (Figure 6G). Rather, the sizes and distribution of the reaction products is the same (Figure 6G). If A3G inhibited RT in the elongation stage, i.e., processivity, we would expect to observe the same amount of total primer extension in the presence or absence of A3G, but that extension products would be shorter in the presence of A3G than in its absence. Instead, we observed that A3G slows down RT initiation as evidenced by less overall primer extension (Figure 6E–F), but does not inhibit formation of full length products, i.e., processivity of RT (Figure 6G).

That we found the RNA binding abilities of A3G and RT to be of importance in the A3G-mediated inhibition of RT polymerization is in contrast to the report by Wang *et al.* [29] that proposed an A3G-RT interaction is responsible. Although the article by Wang *et al.* [29] posits that the A3G-RT interaction is required, data relied on an A3G sequence derived peptide that could block the effect of A3G on RT-mediated polymerization. This raises the question of whether the peptide, which included the dimerization residues of A3G, was binding to RT or A3G [29]. The peptide could have bound A3G and blocked its oligomerization, and as a result affected template binding affinity [16] or the peptide could have blocked A3G from binding RT but could not exert an inhibitory effect on RT because other secondary interactions are required from the full length A3G protein [29]. These possibilities were not clarified [29]. In combination with our binding data (Table 1 and Figure 4), the physical interaction of A3G and RT does not appear to be a mechanism by which A3G blocks RT polymerization. Further, RT molecules in a virion (100 estimated molecules) far exceed the estimated number of A3G molecules in a virion (4 to 7 molecules of A3G in the absence of Vif) [24], [39], which would potentially make a mechanism based on an RT-A3G interaction ineffective.

Overall our study suggests that A3G decreases the rate of initiation of primer extension by RT, but is unlikely to cause complete inhibition of DNA synthesis in virions. However, the inhibition of RT polymerization does not necessarily need to be an “all or nothing” phenomenon. This is supported by Gillick *et al.* that conducted studies in primary cell lines or 293T cells with near physiological levels of A3G and found that A3G induced mutagenesis of HIV-1 in addition to decreasing the formation of viral transcripts [27]. Since the decreased rate of DNA synthesis by RT would leave the synthesized (−)DNA exposed after RNase H activity removed the (+)RNA for longer periods of time, this would enable more access of A3G to the (−)DNA. Given that the number of mutations induced by A3G is dependent on the time that the (−)DNA is single stranded [9], [52], [53], the inhibition of RT polymerization may primarily serve as a way of increasing the amount of deamination rather than inhibiting (−)DNA accumulation *per se*.

## Supporting Information

Table S1 **Primers and substrates.**
(PDF)
