# Supplementary material for: Retroviral Restriction Factor APOBEC3G Delays the Initiation of DNA Synthesis by HIV-1 Reverse Transcriptase
Source: PLoS One. 2013 May 23;8(5):e64196. doi: 10.1371/journal.pone.0064196 (PMC3662766; doi:10.1371/journal.pone.0064196)
Supplement: Table S1 — Primers and substrates. (PDF) [file pone.0064196.s001.pdf]

SUPPORTING INFORMATION

Retroviral Restriction Factor APOBEC3G Delays the Initiation of DNA Synthesis by HIV-1 Reverse Transcriptase

Madison B. Adolph, Jonathon Webb, and Linda Chelico

Department of Microbiology & Immunology, University of Saskatchewan, Saskatoon, Saskatchewan, S7N 5E5, Canada.

Address correspondence to: Linda Chelico, 107 Wiggins Road, Department of Microbiology & Immunology, University of Saskatchewan, Canada, S7N 5E5. Fax: 306-966-4298; E-mail: linda.chelico@usask.ca.

Table S1. Primers and substrates.

Name	Sequence
PBS template (Forward)	TGT TAG GAC TCT GGT AAC TAG AG
PBS template (Reverse)	GTC CCT ATT AAC TTT CGC TTT CAA G
protease template (Forward)	ATA GGA GGA CAA CTG AAA GAA GC
protease template (Reverse)	GAT AAA ACC TCC AAT TCC CCC TAT C
E259Q SDM (Forward)	CCT TGA AGG CCG CCA TGC ACA GCT GTG CTT CCT GGA CGT ATT C
E259Q SDM (Reverse)	GAA TAC GTC CAG GAA GCA CAG CTG TGC ATG GCG GCC TTC AAG G
PBS primer	GUC CCU GUU CGG GCG CCA
PBS + 6 primer	GUC CCU GUU CGG GCG CCA CTG CTA
protease primer	GAT AAA ACC TCC AAT TCC CC
Fam-PBS primer	Fam-GUC CCU GUU CGG GCG CCA
Fam-PBS + 6 primer	Fam-GUC CCU GUU CGG GCG CCA CTG CTA
Fam-protease primer	Fam-GAT AAA ACC TCC AAT TCC CC
PBS template	UGU UAG GAC UCU GGU AAC UAG AGA UCC CUC AGA UCA CUC UAG ACU GAG UAA AAA UCU CUA GCA G**UG GCG CCC GAA CAG GGA C**UU GAA AGC GAA AGU UAA UAG GGA C
Protease template	AUA GGA GGA CAA CUG AAA GAA GCU CUA UUA GAU ACA GGA GCA GAU GAU ACA GUA UUA GAA GAU AUA AAU UUG CCA GGG AAA UGG AAA CCA AAA AUG AUA GGG GGA AUU GGA GGU UUU AUC

Abbreviations: Fam is fluorescein.
